# Identifying implementation bottlenecks for maternal and newborn health interventions in rural districts of the United Republic of Tanzania

**DOI:** 10.2471/BLT.14.141879

**Published:** 2015-04-22

**Authors:** Ulrika Baker, Stefan Peterson, Tanya Marchant, Godfrey Mbaruku, Silas Temu, Fatuma Manzi, Claudia Hanson

**Affiliations:** a Department of Public Health Sciences, Global Health–Health Systems and Policy Research, Karolinska Institutet, Widerströmska huset, Tomtebodavägen 18A, 17177, Stockholm, Sweden.; b Department of Women’s and Children’s Health, International Maternal and Child Health (IMCH), Uppsala University, Uppsala, Sweden.; c Department of Disease Control, London School of Hygiene & Tropical Medicine, London, England.; d Ifakara Health Institute, Dar es Salaam, United Republic of Tanzania.

## Abstract

**Objective:**

To estimate effective coverage of maternal and newborn health interventions and to identify bottlenecks in their implementation in rural districts of the United Republic of Tanzania.

**Methods:**

Cross-sectional data from households and health facilities in Tandahimba and Newala districts were used in the analysis. We adapted Tanahashi’s model to estimate intervention coverage in conditional stages and to identify implementation bottlenecks in access, health facility readiness and clinical practice. The interventions studied were syphilis and pre-eclampsia screening, partograph use, active management of the third stage of labour and postpartum care.

**Findings:**

Effective coverage was low in both districts, ranging from only 3% for postpartum care in Tandahimba to 49% for active management of the third stage of labour in Newala. In Tandahimba, health facility readiness was the largest bottleneck for most interventions, whereas in Newala, it was access. Clinical practice was another large bottleneck for syphilis screening in both districts.

**Conclusion:**

The poor effective coverage of maternal and newborn health interventions in rural districts of the United Republic of Tanzania reinforces the need to prioritize health service quality. Access to high-quality local data by decision-makers would assist planning and prioritization. The approach of estimating effective coverage and identifying bottlenecks described here could facilitate progress towards universal health coverage for any area of care and in any context.

## Résumé

**Objectif:**

Estimer la couverture effective des interventions de santé maternelle et néonatale et identifier les goulots d'étranglement dans leur mise en œuvre, dans des districts ruraux de République-Unie de Tanzanie.

**Méthodes:**

Des données transversales, obtenues auprès de ménages et d'établissements de santé dans les districts de Tandahimba et Newala, ont été utilisées dans l'analyse. Nous avons adapté le modèle de Tanahashi pour estimer la couverture des interventions lors de leurs différentes étapes conditionnelles et identifier les goulots d'étranglement en termes d'accès, de disponibilité opérationnelle des centres de santé et de pratiques cliniques. Les interventions étudiées portaient sur le dépistage de la syphilis et de la prééclampsie, l'utilisation du partogramme, la prise en charge active du troisième stade du travail et les soins post-partum.

**Résultats:**

La couverture effective s'est révélée faible dans les deux districts : entre seulement 3 % pour les soins post-partum dans le district de Tandahimba et 49 % pour la prise en charge active du troisième stade du travail dans le district de Newala. Dans le district de Tandahimba, le principal goulot d'étranglement identifié pour la plupart des interventions concerne la disponibilité opérationnelle des centres de santé, tandis que pour le district de Newala, il s'agit de l'accès. Les pratiques cliniques ont aussi largement entravé le dépistage de la syphilis dans les deux districts.

**Conclusion:**

La faible couverture effective des interventions de santé maternelle et néonatale dans les districts ruraux de République-Unie de Tanzanie renforce la nécessité de donner la priorité à la qualité des services de santé. Or, si les décideurs pouvaient accéder à des données locales de grande qualité, cela permettrait d'améliorer les activités de planification et de hiérarchisation. L'approche d'estimation de la couverture effective et d'identification des goulots d'étranglement qui est décrite ici pourrait favoriser les progrès vers la couverture sanitaire universelle, dans tous les domaines de soin et tous les contextes.

## Resumen

**Objetivo:**

Estimar la cobertura efectiva de intervenciones de salud maternal y neonatal e identificar obstáculos en su implementación en distritos rurales de la República Unida de Tanzania.

**Métodos:**

En el análisis se utilizaron datos transversales de hogares y centros sanitarios de los distritos de Tandahimba y Newala. Se adaptó el modelo de Tanahashi para estimar la cobertura de intervención en etapas condicionales y para identificar obstáculos en la implementación en el acceso y la disponibilidad de los centros sanitarios y la práctica clínica. Las intervenciones estudiadas fueron las pruebas para la sífilis y la preeclampsia, el uso del partograma, la gestión activa de la tercera etapa del parto y el postparto.

**Resultados:**

La cobertura resultó ser poco eficaz****en ambos distritos, desde sólo un 3% en el postparto en Tandahimba hasta un 49% en la gestión activa de la tercera etapa del parto en Newala. En Tandahimba, la disponibilidad de los centros sanitarios era el obstáculo más importante en la mayoría de intervenciones, mientras que en Newala el mayor obstáculo era el acceso. La práctica clínica resultó ser otro obstáculo para las pruebas de sífilis en ambos distritos.

**Conclusión:**

La ineficiente cobertura de las intervenciones de salud maternal y neonatal en distritos rurales de la República Unida de Tanzania reafirma la necesidad de priorizar la calidad del servicio sanitario. Si quienes toman las decisiones pudieran acceder a datos locales de alta calidad, la planificación y la priorización mejorarían. El enfoque de estimar la cobertura efectiva e identificar los obstáculos aquí descritos podría facilitar el avance hacia una cobertura sanitaria universal en cualquier área de la atención médica y en cualquier contexto.

## ملخص

**الغرض:**

تقدير التغطية الفعالة للتدخلات الصحية للأمهات والأطفال حديثي الولادة وتحديد عوامل الاختناق التي تؤثر على تنفيذها في المناطق الريفية في جمهورية تنزانيا المتحدة.

**الطريقة:**

تم استخدام بيانات مقطعية عرضية من الأسر والمرافق الصحية في مناطق تانداهيمبا ونيوالا في التحليل. وقمنا بتعديل نموذج تاناهاشي لتقدير تغطية التدخل في المراحل الشَرطية، والوقوف على عوامل الاختناق المؤثرة على التنفيذ من حيث إتاحة سبل الوصول للخدمات، ومدى استعداد المرافق الصحية، والممارسة السريرية. وكانت التدخلات التي تمت دراستها هي الزهري، وفحص مقدِّمات الارتعاج، واستخدام مُخطَّط المخاض، والإدارة الفعالة للمرحلة الثالثة من المخاض والرعاية التالية للولادة.

**النتائج:**

كانت التغطية الفعالة منخفضة في المنطقتين، وتتراوح نسبتها ما بين ‏3‏‏% فقط للرعاية التالية للولادة في تانداهيمبا إلى 49‏‏‏% للإدارة الفعالة للمرحلة الثالثة من المخاض في نيوالا. في تانداهيمبا، كان استعداد المرافق الصحية هو عامل الاختناق الأكبر بالنسبة لمعظم التدخلات، بينما في نيوالا، كان تيسير سبل الوصول إلى الخدمات هو عامل الاختناق الأكبر. وكانت الممارسة السريرية تمثل عاملاً آخر للاختناق بالنسبة لفحص الزهري في المنطقتين.

**الاستنتاج:**

إن سوء التغطية الفعالة للتدخلات الصحة للأمهات والأطفال حديثي الولادة في المناطق الريفية في جمهورية تنزانيا المتحدة يعزز الحاجة إلى إعطاء الأولوية لجودة الخدمات الصحية. وقد يفيد تيسير سبل اطلاع صنّاع القرار على بيانات محلية عالية الجودة في التخطيط وتحديد الأولويات. ويمكن للأسلوب المتبع والموضح في هذه النشرة لتقدير التغطية الفعالة وتحديد عوامل الاختناق أن يسهل من إحراز التقدم نحو التغطية الصحية الشاملة لأي مجال من مجالات الرعاية وفي أي سياق.

## 摘要

**目的:**

旨在估计孕产妇和新生儿卫生干预的有效覆盖率，并确定他们在坦桑尼亚联合共合作境内的农村地区的实施瓶颈。

**方法:**

分析中采用了从 Tandahimba 和 Newala 地区的家庭和卫生设施处采集的横截面数据。我们采用了 Tanahashi 的模式来估计各个条件阶段的干预覆盖率，并确定在使用、卫生设施可用性和临床实践方面的实施瓶颈。研究的干预措施为梅毒和子痫前期筛选、产程图使用、分娩第三期的积极管理以及产后护理。

**结果:**

两个地区的有效覆盖率都很低，Tandahimba 地区内产后护理仅为 3%，Newala 地区内分娩第三期的积极管理为 49%。在 Tandahimba 地区，卫生设施可用性是大部分干预措施的最大瓶颈，而在 Newala 地区是可以获取的。针对两个地区的梅毒筛选，临床实践为另一大瓶颈问题。

**结论:**

坦桑尼亚联合共和国境内的农村地区对孕产妇和新生儿卫生干预的有效覆盖率差这一点加强了优先发展卫生服务质量这一需求。由决策制定者获取高质量的本地数据将有助于进行规划和排定优先级。估计有效覆盖率并确定此处所述之瓶颈问题的方法可促进全民卫生覆盖计划在任何护理领域和任何环境中的发展。

## Резюме

**Цель:**

Оценка эффективности охвата мероприятий по оказанию медико-санитарной помощи матери и ребенку, определение препятствий для ее практической реализации в сельской местности Объединенной Республики Танзания.

**Методы:**

В ходе анализа использовались данные перекрестного исследования домашних хозяйств и лечебных учреждений в округах Тандахимба и Невала. Для оценки мероприятий по оказанию медико-санитарной помощи на условных этапах, определения затруднений доступа медико-санитарной помощи, а также готовности лечебных учреждений и клинической практики была адаптирована модель Танахаши. Исследовались мероприятия по скрининговому обследованию сифилиса и преэклампсии, использованию партографа, активному ведению пациентки на третьей стадии родов и послеродовому уходу.

**Результаты:**

Эффективность охвата оказалась низкой в обоих округах исследования. Она колебалась от 3% для послеродового ухода в Тандахимба до 49% для активного ведения пациентки на третьей стадии родов в Невала. Готовность лечебных учреждений в Тандахимба стала самым большим препятствием для реализации большинства мер по охране здоровья, в то время как в Невала был затруднен доступ к медико-санитарной помощи. В обоих округах большим препятствием для скринингового обследования сифилиса являлась клиническая практика.

**Вывод:**

Низкая эффективность охвата мероприятиями по оказанию медико-санитарной помощи матери и ребенку в сельской местности Объединенной Республики Танзания подчеркивает необходимость сосредоточить внимание на качестве медицинского обслуживания. Доступ к достоверным локальным данным о принятии решений может способствовать процессу планирования и определения приоритетов. Описанный в данной статье подход к оценке эффективности охвата и определению проблемных мест может способствовать обеспечению всеобщего охвата мероприятий по оказанию медико-санитарной помощи населению в любой области здравоохранения и в любом контексте.

## Introduction

Although maternal and newborn mortality has been substantially reduced worldwide in recent years, progress has been uneven. In sub-Saharan Africa, few countries are on track to meet Millennium Development Goals (MDGs) 4 and 5 on child mortality and maternal heath, respectively.^1^^,^^2^ Weak health systems have failed to achieve effective coverage of key interventions – they have been unable to reach mothers and newborns with interventions that were implemented as intended, with a potential impact on mortality.^3^ A bottleneck has been defined as “that component of a system that limits the overall performance or capacity of the system.”^4^ Consequently, unless bottlenecks are targeted, efforts to strengthen health systems will have little effect.^4^ Since identifying bottlenecks in health service delivery can help in setting priorities, it is an important area of research in maternal and newborn health and in attempts to strengthen district health systems.^1^^,^^5^Bottlenecks in implementation can be due to limited access, for geographical, financial or sociocultural reasons. Poor readiness of health-care facilities due to, for example, a lack of human resources, drugs or equipment and suboptimal clinical practice, such as failure to adhere to evidence-based clinical guidelines can also cause bottlenecks.^4^^,^^6^^–^^9^

Previously, monitoring improvements in maternal health focused primarily on the service use: for example, the proportion of mothers attending antenatal care or giving birth in a health facility. Although these are important indicators, they do not reflect the content or quality of the care provided or the extent to which key interventions are implemented as intended.^2^ Currently, this measurement gap is one element in the discussions on universal health coverage that are taking place as part of the post-MDG agenda, in which the importance of quality-of-care indicators for assessing population coverage is emphasized.^10^^,^^11^

In 1978, Tanahashi described a way of both measuring health service coverage and identifying bottlenecks in implementation.^12^ Since then, his approach has been used and modified by the United Nations Children’s Fund and the World Bank.^13^ Although the coverage measures in Tanahashi’s model both reflect quality of care and reveal implementation bottlenecks, there are limitations. First, the model focuses initially on health service capacity rather than output. Second, the assessments require high-quality data from health management information systems, which are rarely available in low-income settings, particularly for intrapartum interventions and subnational analyses.^14^^–^^16^ These limitations could be overcome by linking household and health facility data,^14^ as has been done previously for malaria care.^17^^,^^18^

Our objectives were to estimate the effective coverage of key maternal and newborn health interventions in rural parts of the United Republic of Tanzania and to identify bottlenecks in implementation. 

## Methods

We used data from an observational, cross-sectional study that was performed in Tandahimba and Newala districts in south-eastern United Republic of Tanzania.^19^ Each district has a population of approximately 200 000 people and is characterized by high maternal and newborn mortality: in 2004–2007, the estimated maternal mortality ratio was 712 per 100 000 live births^20^ and the estimated neonatal mortality rate was 31 per 1000 live births.^21^ Data were collected as part of the EQUIP (Expanded Quality Management Using Information Power) project, which was a collaborative, quality improvement intervention for maternal and newborn care implemented in health facilities and communities in Tandahimba between November 2011 and April 2014.^19^^,^^22^ Continuous household surveys and repeated health facility censuses were conducted to provide feedback on, and monitor the effects of, the EQUIP intervention.^19^ These surveys and censuses were also carried out in Newala, an adjacent district where the intervention was not implemented. Our study involved EQUIP household data collected between November 2011 and December 2012 and health facility data from a census conducted between April and July 2012. Data were collected before full implementation of the EQUIP project and, therefore, before quality improvements due to the intervention would have been expected.

The household survey involved continuous cluster sampling. Each month, 10 household clusters (i.e. subvillages) were selected, with the probability of selection being proportional to the population size in the district. Within each cluster, 30 households were selected by simple random sampling. Interviews were held with the head of the household and with all resident women aged 13 to 49 years and a special interview module was used for women who had recently had a live birth. Questions on care-seeking, treatment and outcomes during pregnancy and childbirth were included.^19^ We included only women who had had a live birth in the 12 months before the survey.

The health facility census, which was repeated every four months, used a checklist to assess readiness. In addition, interviews were conducted with the head of each facility on the services offered and the routine care provided. To obtain information on clinical practice during intrapartum care, the health worker who attended the most recent delivery in the facility was identified and interviewed using a last event module. Questions focused on the actions taken before, during and after the most recent delivery attended and the care provided to the mother. Since health workers were not prompted during the interviews, only actions they remembered or mentioned were recorded.^19^

The study received ethical approval from the Ifakara Health Institute Institutional Review Board in Dar es Salaam (IHI/IRB/ No: 30–2012) and the National Institute for Medical Research of the United Republic of Tanzania (NIMR/HQ/R.8a/Vol. IX/1704). Written consent was obtained from all participants in the household and health facility interviews. Throughout the EQUIP project, results were shared regularly with community members, health workers and district management.

We investigated five key maternal and newborn health interventions: (i) syphilis screening; (ii) pre-eclampsia screening; (iii) use of a partograph to monitor labour; (iv) active management of the third stage of labour; and (v) postpartum care in a health facility. These interventions have all been shown to be associated with a decline in mortality when implemented as intended and the World Health Organization regards them as key interventions that should be delivered through health facilities.^23^

### Coverage and bottlenecks

**Fig. 1:**
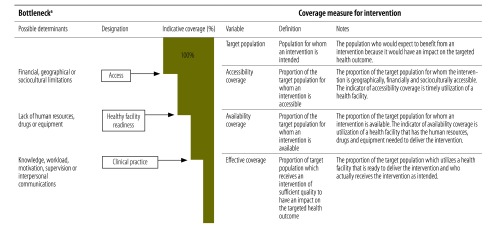
Coverage measures and bottlenecks in the implementation of maternal and newborn health interventions

^a^ The magnitude of the bottleneck is the attrition in coverage between one stage of the implementation pathway and the next.

We adapted Tanahashi’s original model^12^ to estimate the actual coverage of an intervention at the different conditional stages of its implementation and, subsequently, to identify bottlenecks between these stages. We call this model the implementation pathway (Fig. 1). It includes three coverage stages: (i) accessibility coverage, which is the proportion of the target population for whom an intervention is accessible; (ii) availability coverage, which is the proportion for whom an intervention is available; and (iii) effective coverage, which is the proportion who receive an intervention of sufficient quality to affect the targeted health outcome (Fig. 1). For each intervention, coverage was calculated by dividing**the number of individuals who satisfy the conditions for implementation at a particular stage by the target population.

Each stage is conditional on the preceding stage. Table 1 outlines how coverage measures for each intervention were estimated. Depending on the intervention, the target population was defined normatively as either all women who were pregnant or all women who gave birth during the study period.

**Table 1:** Target populations and coverage measures for maternal and newborn health interventions in the United Republic of Tanzania

Variable	Definition of target population or coverage measure for the intervention				
	Syphilis screening	Pre-eclampsia screening	Use of partograph to monitor labour	Active management of the third stage of labour	Postpartum care in a health facility
**Target population^a^**	All women during pregnancy	All women during pregnancy	All women during childbirth	All women during childbirth	All women after childbirth
**Coverage measure in implementation pathway**					
Accessibility coverage	Proportion attending antenatal care at least once during pregnancy	Proportion attending antenatal care at least three times during pregnancy	Proportion giving birth in a health facility	Proportion giving birth in a health facility	Proportion giving birth in a health facility
Availability coverage	Proportion attending antenatal care in a health facility with a syphilis test available	Proportion attending antenatal care in a health facility with a sphygmomano-meter available	Proportion giving birth in a health facility with a partograph available	Proportion giving birth in a health facility with sterile syringes and needles and oxytocin or ergometrine available	Proportion giving birth in a health facility offering postpartum care with iron supplements available
Effective coverage	Proportion who satisfy the definition for availability coverage and who report having a blood test and receiving a test result for syphilis	Proportion who satisfy the definition for availability coverage and who report having their blood pressure checked	Proportion who satisfy the definition for availability coverage and who used a facility in which a health worker reported using a partograph during the last delivery attended	Proportion who satisfy the definition for availability coverage and who used a facility in which a health worker reported giving an oxytocic agent during the last delivery attended	Proportion who satisfy the definition for availability coverage and who report being checked within 48 hours of delivery

^a^ The target population is the denominator for all coverage measures. For each stage along the implementation pathway, the coverage measure is conditional on the preceding stage.

One difference between Tanahashi’s original model and our implementation pathway is that the first stage is accessibility coverage rather than availability coverage (Fig. 1). We reasoned that, if an intervention is actually to be available to its target population, that population first needs to have access to a health facility where it could be delivered. Consequently, the indicator used for accessibility coverage is the utilization of health services: in our study, this meant either attending antenatal care or giving birth at a health facility. Information on these two indicators was derived from the household survey. Acceptability coverage as defined in Tanahashi’s original model was considered a determinant of accessibility rather than a separate stage of implementation.

In our implementation pathway, availability coverage was defined as the proportion of mothers who used a health facility that was able to deliver the intervention (i.e. sufficient human resources, drugs and equipment were available). We estimated availability coverage by multiplying indicators of utilization from the household survey by indicators of health facility readiness; both indicators were stratified by health facility level (i.e. hospital, health centre or dispensary). For example, the proportion of mothers who used dispensaries was multiplied by the proportion of dispensaries able to deliver the intervention. The stratified results were combined to derive the overall availability coverage for each intervention.

Effective coverage in our implementation pathway – the final stage of implementation – was defined as the proportion of mothers who used a health facility that was ready to deliver the intervention and who actually received the intervention. As for availability coverage, the analysis was stratified by health facility level. Indicators of antenatal and postpartum interventions were derived from interviews with mothers and indicators of intrapartum interventions were derived from health workers’ reports.

Bottlenecks in implementation were identified from the absolute attrition in coverage between one stage and the next. Although bottlenecks could have many possible underlying determinants, we designated them as bottlenecks in access, health facility readiness or clinical practice (Fig. 1).

The sample size for the EQUIP household survey was such that coverage of key maternal and newborn health interventions could be estimated with 80% power at the district level every four months. All statistical analyses were performed using Stata version 12 (StataCorp. LP, College Station, United States of America). Proportions and confidence intervals (CI) for indicators from the household survey were computed using the “svy” command to adjust for the effect of clustering. CIs were not computed for the coverage measures because, apart from accessibility coverage, all measures were derived from a combination of survey and census data. Throughout, missing values were treated as indicating that the intervention had not been implemented. Missing values accounted for 0 to 8% of data for all indicators apart from syphilis test availability, for which 19% of values were missing. No significant change in coverage measures was detected in sensitivity analyses.

## Results

Our analysis included data from household surveys on 772 women, from interviews with 70 health workers and from a health facility census of 60 facilities (Table 2). Health facility utilization is shown in Table 3 and estimates of coverage indicators are presented in Table 4 for individual care received, in Table 5 for health facility readiness and in Table 6 for clinical practice.

**Table 2:** Household survey,^a^ November 2011 to December 2012, and health facility census and health worker interviews, April to July 2012, United Republic of Tanzania

Entity or individuals assessed	No.		
	Tandahimba district	Newala district	Total
Households interviewed in the survey	3436	3494	6930
Women of reproductive age (i.e. 13–49 years) interviewed	3196	2979	6175
Women who had had a live birth in the 12 months before the survey	400	372	772
Health facilities covered by the census	32	28	60
Health workers interviewed	39	31	70

^a^ The household survey was carried out as part of the EQUIP (Expanded Quality Management Using Information Power) project.

**Table 3:** Health facility utilization^a^ for maternal and newborn health interventions, 2011–2012, United Republic of Tanzania

Intervention	Women with a live birth in the previous 12 months, No. (%)								
	Tandahimba district					Newala district			
	Total	Hospital	Health centre	Dispensary		Total	Hospital	Health centre	Dispensary
Attended antenatal care	396 (100)	59 (15)	52 (13)	285 (72)		372 (100)	56 (15)	48 (13)	268 (72)
Gave birth in a health facility	240 (100)	120 (50)	29 (12)	91 (38)		212 (100)	121 (57)	11 (5)	80 (38)

^a^ Information on utilization was derived from household survey interviews with mothers who had had a live birth in the previous 12 months.

**Table 4:** Individual indicators of coverage of maternal and newborn health interventions, 2011–2012, United Republic of Tanzania

Indicator of coverage of an intervention^a^	Women with a live birth in the previous 12 months			
	Tandahimba district (*n* = 400)		Newala district (*n* = 372)	
	No.	% (95% CI)	No.	% (95% CI)
Attended antenatal care ≥ 1 time	396	99 (98–100)	372	100 (99–100)
Attended antenatal care ≥ 3 times	312	78 (75–82)	246	66 (61–71)
Gave birth in a health facility	240	60 (55–65)	212	57 (51–63)
Gave blood for any test during antenatal care	328	82 (79–86)	350	94 (91–96)
Received a syphilis test result during antenatal care	100	25 (21–30)	97	26 (20–31)
Had blood pressure checked during antenatal care	220	55 (50–61)	223	60 (54–67)
Were checked within 48 hours of delivery	84	21 (17–25)	67	18 (13–23)

CI: confidence interval.

^a^ Indicators were derived from household survey interviews with mothers who had had a live birth in the previous 12 months.

**Table 5:** Health facility readiness indicators for estimating coverage of maternal and newborn health interventions, 2011–2012, United Republic of Tanzania

Indicator of readiness for an intervention^a^	Facilities ready to deliver the intervention, No. (%)								
	Tandahimba district					Newala district			
	All (*n* = 32)	Hospital (*n* = 1)	Health centre (*n* = 3)	Dispensary (*n* = 28)		All (*n* = 28)	Hospital *(n =* 1)	Health centre (*n* = 2)	Dispensary *(n =* 25)
Syphilis test in stock	13 (41)	1 (100)	1 (33)	11 (39)		20 (71)	1 (100)	2 (100)	17 (68)
Sphygmomanometer in stock	12 (38)	1 (100)	2 (67)	9 (32)		17 (61)	1 (100)	2 (100)	14 (56)
Blank partographs in stock	13 (41)	0 (0)	3 (100)	10 (36)		16 (57)	1 (100)	2 (100)	13 (52)
Sterile needles in stock	31 (97)	1 (100)	3 (100)	27 (96)		28 (100)	1 (100)	2 (100)	25 (100)
Oxytocin or ergometrine in stock	18 (56)	1 (100)	3 (100)	14 (50)		23 (82)	1 (100)	2 (100)	20 (80)
Any iron supplement in stock	16 (50)	0 (0)	1 (33)	15 (54)		21 (75)	0 (0)	2 (100)	19 (76)

^a^ Information on indicators was derived from a health facility census, which recorded commodities physically available on the day of the census.

**Table 6:** Clinical practice indicators of coverage of maternal and newborn health interventions, 2011–2012, United Republic of Tanzania

Indicator of coverage by an intervention^a^	Health workers who reported implementing the intervention, No. (%)								
	Tandahimba district					Newala District			
	All *(n* = 39)	Hospital-based *(n* = 3)	Health centre-based *(n* = 3)	Dispensary-based *(n* = 33)		All *(n* = 31)	Hospital-based *(n* = 2)	Health centre-based *(n* = 2)	Dispensary-based *(n* = 27)
Use of a partograph	27 (69)	2 (67)	3 (100)	22 (67)		25 (81)	1 (50)	2 (100)	22 (81)
Any oxytocic agent administered	23 (59)	2 (67)	2 (67)	19 (58)		26 (84)	2 (100)	2 (100)	22 (81)

^a^ Information on indicators was derived from interviews with health workers who reported on the actions taken during the last delivery attended.

**Fig. 2:**
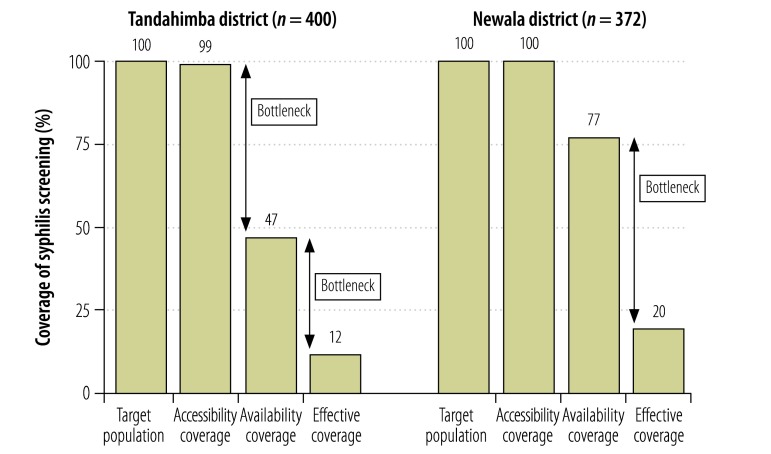
Coverage of and bottlenecks in syphilis screening of pregnant women, 2011–2012, United Republic of Tanzania

Notes: Bottlenecks in access, health facility readiness and clinical practice are indicated for ≥ 30% attrition in coverage.

**Fig. 3:**
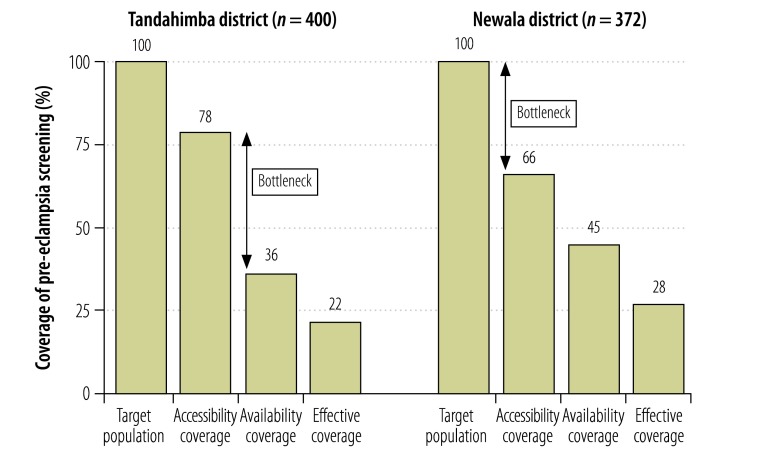
Coverage of and bottlenecks in pre-eclampsia screening of pregnant women, 2011–2012, United Republic of Tanzania

Notes: Bottlenecks in access, health facility readiness and clinical practice are indicated for ≥ 30% attrition in coverage.

Estimated effective coverage of syphilis screening in Tandahimba was 12% despite near universal accessibility coverage (Fig. 2). The largest bottleneck was health facility readiness, which was associated with a 52% reduction in coverage. Clinical practice was another large bottleneck, with an attrition of 35%. In Newala, accessibility coverage was 100% and estimated effective coverage was 20%. Here, clinical practice was the largest bottleneck, causing an attrition of 57%. Estimated effective coverage of pre-eclampsia screening in Tandahimba was 22%, with health facility readiness being the largest bottleneck, causing an attrition of 42% (Fig. 3). In Newala, effective coverage was 28%, with access being the largest bottleneck, causing an attrition of 34%.

**Fig. 4:**
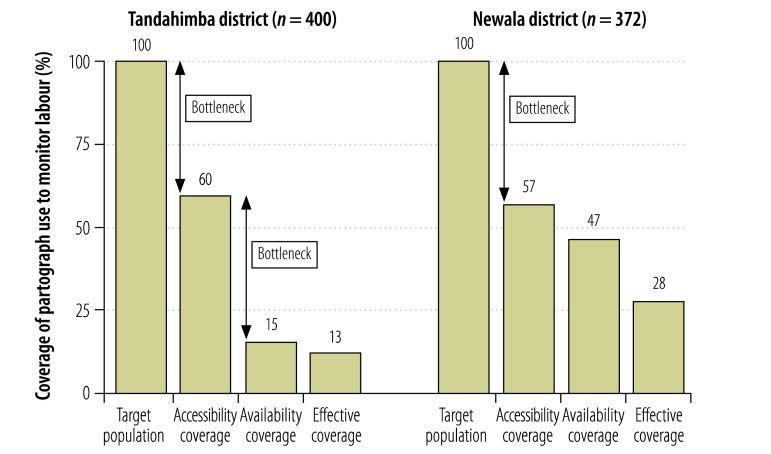
Coverage of and bottlenecks in partograph use for monitoring labour, 2011–2012, United Republic of Tanzania

Notes: Bottlenecks in access, health facility readiness and clinical practice are indicated for ≥ 30% attrition in coverage.

**Fig. 5:**
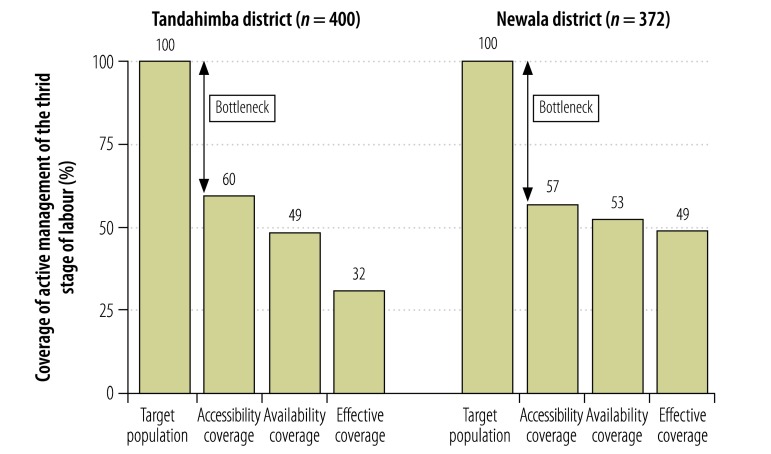
Coverage of and bottlenecks in active management of the third stage of labour, 2011–2012, United Republic of Tanzania

Notes: Bottlenecks in access, health facility readiness and clinical practice are indicated for ≥ 30% attrition in coverage.

Estimated effective coverage of partograph use to monitor labour in Tandahimba was 13% (Fig. 4). Health facility readiness was the largest bottleneck, causing an attrition of 45%, though access was another large bottleneck, with an attrition of 40%. In Newala, estimated effective coverage was 28%, with access being the largest bottleneck, causing an attrition of 43%. Estimated effective coverage of active management of the third stage of labour in Tandahimba was 32%, with access being the largest bottleneck, causing an attrition of 40% (Fig. 5). In Newala, estimated effective coverage was 49%, again with access being the largest bottleneck, causing an attrition of 43%.

**Fig. 6:**
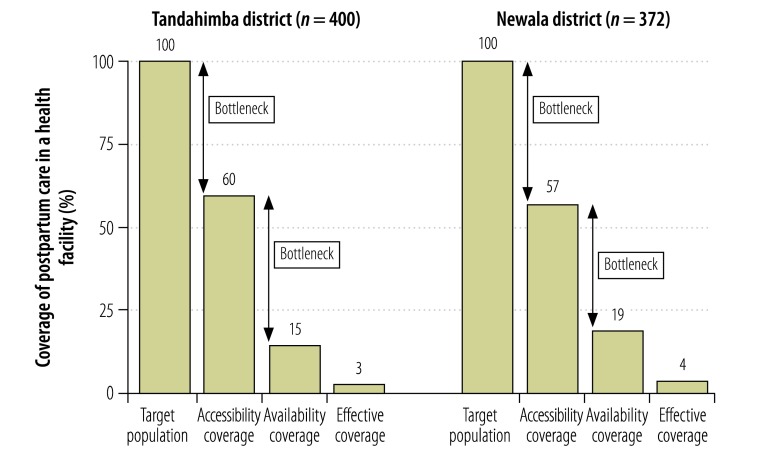
Coverage of and bottlenecks in postpartum care in a health facility, 2011–2012, United Republic of Tanzania

Notes: Bottlenecks in access, health facility readiness and clinical practice are indicated for ≥ 30% attrition in coverage.

Estimated effective coverage of postpartum care in a health facility in Tandahimba was only 3% (Fig. 6). The largest bottleneck was health facility readiness, which was associated with an attrition of 45%. Access was another large bottleneck, with an attrition of 40%. In Newala, effective coverage was also low at 4%, with access being the largest bottleneck, causing an attrition of 43%. Another large bottleneck was health facility readiness, with an attrition of 38%.

Effective coverage was similar in the two districts for all interventions: the difference was 15 percentage points or less, except for active management of the third stage of labour, where it was 17%. The largest implementation bottleneck was the same in the two districts for only one intervention: access to active management of the third stage of labour. However, within districts, the largest bottlenecks were similar across interventions. In Tandahimba, health facility readiness was the largest bottleneck for all interventions apart from active management of the third stage of labour, where access was the largest bottleneck. In Newala, access was the largest bottleneck for all interventions, apart from syphilis screening, where clinical practice was the largest bottleneck.

## Discussion

Effective coverage of key health interventions for mothers and newborns was low in both study districts: it ranged from 3% for postpartum care in a health facility in Tandahimba to 49% for active management of the third stage of labour in Newala. Apart from active management of the third stage of labour, effective coverage was 28% or less for all interventions in the two districts. In Tandahimba, health facility readiness was the largest bottleneck for most interventions, whereas, in Newala, it was access to a health facility. Clinical practice was a substantial bottleneck for syphilis screening in both districts.

Although antenatal care attendance was almost universal and despite a substantial increase in health facility deliveries from 41% in 2007^24^ to around 60% in 2012 in our study, effective coverage of key interventions remained low, which is consistent with previous reports of health system weaknesses in the study area.^25^ Moreover, our findings are consistent with evidence that a focus on access to care alone does not reduce maternal mortality.^26^ More emphasis must be placed on the quality of health services: health facility readiness could be increased by introducing better policies on essential commodities and clinical practice could be enhanced, for example, by quality improvement interventions.^26^^,^^27^

Our findings highlight the complex interaction between the capacity of a health system and its outputs. For example, it has been shown that a lack of drugs and equipment can demotivate staff and undermine good clinical practice, even when drugs and equipment subsequently become available.^9^^,^^25^ This might explain our observation that clinical practice was a bottleneck for syphilis screening even when mothers used health facilities with syphilis tests in stock.

Although the levels of effective coverage were similar in our two study districts, there was a difference in the pattern of bottlenecks, which points to variability in local health system functioning. It is important that the reasons for poor effective coverage are disentangled and targeted and that decision-makers have better access to high-quality data at the district level for use in planning and setting priorities.^5^^,^^13^^,^^25^^,^^28^ Linking data from households and health facilities could produce meaningful estimates of coverage that could help tailor the implementation of interventions in specific contexts.

The study has some limitations. The interventions we analysed were all preventive measures, which made it possible to define the target population at the district level.^23^ We were not able to include interventions such as Kangaroo mother care for premature or underweight newborns or management of postpartum haemorrhage because a large proportion of data on birth weight was missing and few cases of postpartum haemorrhage were recorded. Assessing the impact of effective coverage of an intervention using outcome measures such as deaths or adverse events averted for mothers was beyond the scope of this study.

Another limitation is that our coverage estimates used indicators that reflected only the minimum conditions required for judging completeness of implementation. For example, active management of the third stage of labour was judged to have been carried out if the health worker reported the administration of oxytocic agents; controlled cord traction and uterine massage were not considered.^29^ Clearly the indicators chosen affect the coverage estimates. Indicators could be modified or updated as new evidence on the efficacy of an intervention becomes available. The validity of indicators is a generic concern for all surveys but is especially problematic when mothers themselves report indicators of care related to childbirth.^14^^,^^30^ Consequently, we included health workers’ reports of the actions taken during the most recent delivery they attended as indicators of clinical practice. These reports could have been subject to a social desirability bias that resulted in overreporting: health workers may have reported that an intervention was implemented to make a good impression. On the other hand, since the interview questions were open-ended and respondents were not prompted, it is possible that not all actions taken were reported, which would have given rise to underreporting. In addition, we did not link data from individual mothers with data from health facilities or health worker reports. Consequently, the reliability of our estimates of routine delivery care would be affected by the existence of large variations in health facility readiness or clinical practice between facilities at the same health facility level.

In our study, bottlenecks were identified from the absolute attrition in coverage between one stage and the next. However, relative attrition in coverage may be equally important. Also, the aim of our analysis was to estimate effective coverage at the district level. We could not make inferences about differences between different facility levels because we did not have a sufficiently large sample; for example, there were only two hospitals and five health centres in the two districts. Moreover, the differences in readiness between health facilities shown in Table 5 suggest that bottlenecks may differ between facility levels. Identification of these differences could further aid priority-setting.

In conclusion, effective coverage of health interventions, whether preventive, curative or palliative, is an important output against which the capacity of any health system should be evaluated. Our approach to estimating effective coverage and identifying implementation bottlenecks provides a framework that could help operationalize measurements and track progress towards universal health coverage in all areas of health care.
